# Establishment of an immune-related gene pair model to predict colon adenocarcinoma prognosis

**DOI:** 10.1186/s12885-020-07532-7

**Published:** 2020-11-09

**Authors:** Jihang Luo, Puyu Liu, Leibo Wang, Yi Huang, Yuanyan Wang, Wenjing Geng, Duo Chen, Yuju Bai, Ze Yang

**Affiliations:** 1 Cancer Hospital, Second Affiliated Hospital of Zunyi Medical University, Zunyi City, 563000 Guizhou Province China; 2 Department of Pathology, Second Affiliated Hospital of Zunyi Medical University, Zunyi, Guizhou Province China; 3 Department of Urology, Affiliated Hospital of Zunyi Medical University, Zunyi, Guizhou Province China

**Keywords:** Colon adenocarcinoma, Immune-related gene pairs, Prognosis, TCGA, GEO

## Abstract

**Background:**

Colon cancer is the most common type of gastrointestinal cancer and has high morbidity and mortality. Colon adenocarcinoma (COAD) is the main pathological type of colon cancer, and much evidence has supported the correlation between the prognosis of COAD and the immune system. The current study aimed to develop a robust prognostic immune-related gene pair (IRGP) model to estimate the overall survival of patients with COAD.

**Methods:**

The gene expression profiles and clinical information of patients with colon adenocarcinoma were obtained from the TCGA and GEO databases and were divided into training and validation cohorts. Immune genes were selected that showed a significant association with prognosis.

**Results:**

Among 1647 immune genes, a model with 17 IRGPs was built that was significantly associated with OS in the training cohort. In the training and validation datasets, the IRGP model divided patients into the high-risk group and low-risk group, and the prognosis of the high-risk group was significantly worse (*P*<0.001). Univariate and multivariate Cox proportional hazard analyses confirmed the feasibility of this model. Functional analysis confirmed that multiple tumor progression and stem cell growth-related pathways were upregulated in the high-risk groups. Regulatory T cells and macrophages M0 were significantly highly expressed in the high-risk group.

**Conclusion:**

We successfully constructed an IRGP model that can predict the prognosis of COAD, providing new insights into the treatment strategy of COAD.

**Supplementary information:**

**Supplementary information** accompanies this paper at 10.1186/s12885-020-07532-7.

## Background

According to the latest GLOBOCAN [1] report, colorectal cancer (CRC) is the third most commonly diagnosed cancer worldwide (10.2%) and has the second-highest mortality rate (9.2%). Approximately 145,600 new colorectal cancer cases occur each year in the United States, among which 101,420 cases are colon cancer, and the remainder is rectal cancer [2]. In recent years, colon cancer mortality has continued to rise in many countries with limited resources and health infrastructure, particularly in South America and Eastern Europe [3]. Colon adenocarcinoma (COAD) is the primary pathological type of colon cancer. Surgery combined with postoperative chemotherapy is currently the main treatment for COAD. However, the survival of COAD has improved due to the continuous advancement of surgical technology. However, postoperative recurrence and chemotherapy resistance remain two major obstacles to the long-term survival of patients [4–6].

With the development of high-throughput omics, various omics techniques, such as whole-genome sequencing, epigenomics, and proteomics, have been applied to study COAD [7–10]. Increasing evidence has shown that COAD is not a consistent disease type but a molecularly heterogeneous disease comprising a series of genetic changes [11]. Tumor heterogeneity can alter the tumor growth rate, invasive ability, sensitivity to drugs, prognosis and other aspects, making it one of the main obstacles affecting tumor treatment [12, 13]. Therefore, dividing patients with COAD into different risk groups based on gene expression profiles helps to predict the risk of tumor progression or metastasis and recurrence and is a necessary prerequisite for proper individualized treatment [14–16].

There is increasing evidence that the immune system plays an important role in the occurrence and development of cancer [17–19]. For example, Salem M [20] found that disrupting the cell surface receptor glycoprotein-A repetitions predominant (GARP) on activated regulatory T (Treg) cells reduces immune tolerance and the development of colon cancer. In recent years, a method based on the relative ranking of gene expression levels was proposed to eliminate the shortcomings of data standardization and scaling in gene expression data processing, achieving reliable results in various studies [21, 22]. The present study selected immune genes that are significantly associated with the prognosis of COAD. Next, we integrated these genes to construct an immune-related gene pair (IRGP) risk model and verified its feasibility as a prognostic marker for COAD.

## Methods

### Sources of colon adenocarcinoma patients

The data analyzed in this study were all obtained from public databases. The training cohort datasets were downloaded from TCGA (https:*//*tcga*-*data.nci.nih.gov*/*tcga) [23], and the validation datasets were obtained from GEO (https://www.ncbi.nlm.nih.gov/geo/). The training cohort datasets included clinical datasets (*n* = 452), transcriptome datasets (*n* = 449), and verification datasets from GSE39582 (*n* = 585) [24] and GSE17538 (*n* = 244) [25].

### Data processing

The human General Transfer Format (hunman.gtf) from Ensemble (https://www.ensembl.org/index.html) [26] was downloaded, and the TCGA data were annotated using Perl language [27]. The chip data file (GSE39582 and GSE17538) was preprocessed using Perl language through the annotation file of the GPL570 platform. Using the above operations, all the gene probe IDs were converted to corresponding gene symbols. To analyze the correlation between the IRGP signature and prognosis in COAD, only patient data containing complete overall survival (OS) were selected.

### Establishment of the prognostic immune-related gene pair (IRGP) model

We downloaded a list of immune-related genes (IRGs) from IMMPORT (https://www.immport.org/) [28], a website with open access to immunoassay data for translation and clinical research. Next, the R language [29] limma package (version 3.42.2) was used to control the list to screen out the IRGs in the downloaded TCGA transcriptome data. To further select valuable IRGPs, we measured and stored IRGs with a relatively high variation on all the platforms in this study (as determined by the median absolute deviation (MAD) > 0.5) [30]. The expression levels of IRGs in each sample in the transcriptome, GSE39582 and GSE17538 were compared in pairs to form each IRGP according to a previously validated method [22]. Specifically, in the pairwise comparison of each sample, if the expression level of the first gene is greater than that of the second gene, the output is 1; otherwise, it is 0. Samples with a ratio of 0 and 1 less than 20% were deleted to retain gene pairs that may be related to survival. These IRGPs were merged with the survival time of the clinical data downloaded by the corresponding platform to evaluate the correlation between each IRGP in the training dataset and overall survival rate of the patient. Based on previous reports [31, 32], we used the R language survival software package (version 3.1–11) to perform univariate Cox regression analysis and *P* < 0.001 to screen the effective IRGPs in the TCGA data. From these IRGPs, we used R language for Lasso Cox proportional hazards regression (glmnet software package, version 3.0–2) to construct the risk score, and the final prognostic model was defined using 17 gene pairs. Finally, in the training cohort, we set the overall survival to 5 years and constructed the time-dependent receiver operating characteristic (ROC) curve (survivalROC, version 1.0.3) to determine the best cutoff value for the risk score and divide patients into low-risk and high-risk groups accordingly.

### Further validation of the model

Using the R package survival and survminer (version 0.4.6), Kaplan–Meier plots were applied to establish survival curves for the high-risk and low-risk groups in the training and verification cohorts. The differences in the survival curves were analyzed using the log-rank test. Cox proportional hazards analysis was used for univariate and multivariate analyses to assess the effect of the risk score and other clinical factors.

### Gene expression profiles (GEPs) of immune cell infiltration in tumors

We used CIBERSORT [33] to infer the relative abundance of tumor-infiltrating immune cells in different risk groups. CIBERSORT estimated the putative proportion of infiltrating immune cells using a reference set with 22 sorted immune cell subtypes for each sample in the training cohort and validation cohorts. Monte Carlo sampling was used in CIBERSORT to calculate the *P*-value of the deconvolution of each sample to provide the estimated confidence. The permutation is set to greater than 100, and the corresponding *P*-value is generated.

### Gene set enrichment analysis (GSEA)

The chemical and genetic perturbation analysis-related documents involved in the study were downloaded from the Molecular Signature Database (MSigDB C2, version 7.1) (https://www.gsea-msigdb.org/gsea/datasets.jsp). GSEA [34] was performed using the R package fgsea (version 1.12.0) with default parameters. A log 2-fold change was made between GEPs in the high-risk vs low-risk groups. The difference in the gene sets between the high- and low-risk groups was compared. Differences with an FDR-adjusted *P* < 0.05 were defined as significant.

### Statistical analysis

For all the above tests, a *P*-value less than 0.05 denoted the presence of a statistically significant difference. Statistical significance was indicated as follows: *P < 0.05, **P < 0.01, ***P < 0.001.

## Results

### Construction of the prognostic IRGP model

**Fig. 1:**
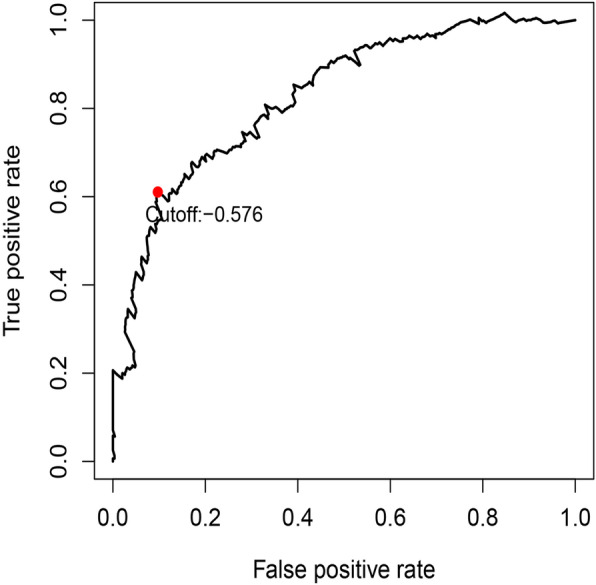
Time-dependent ROC curve for IRGPs risk model in the training cohort. Risk score of − 0.576 which was used as cut-off value for the model to stratify patients into high risk group or low risk group. Abbreviations: ROC, receiver operating characteristic; IRGPs, immune-related gene pairs

**Fig. 2:**
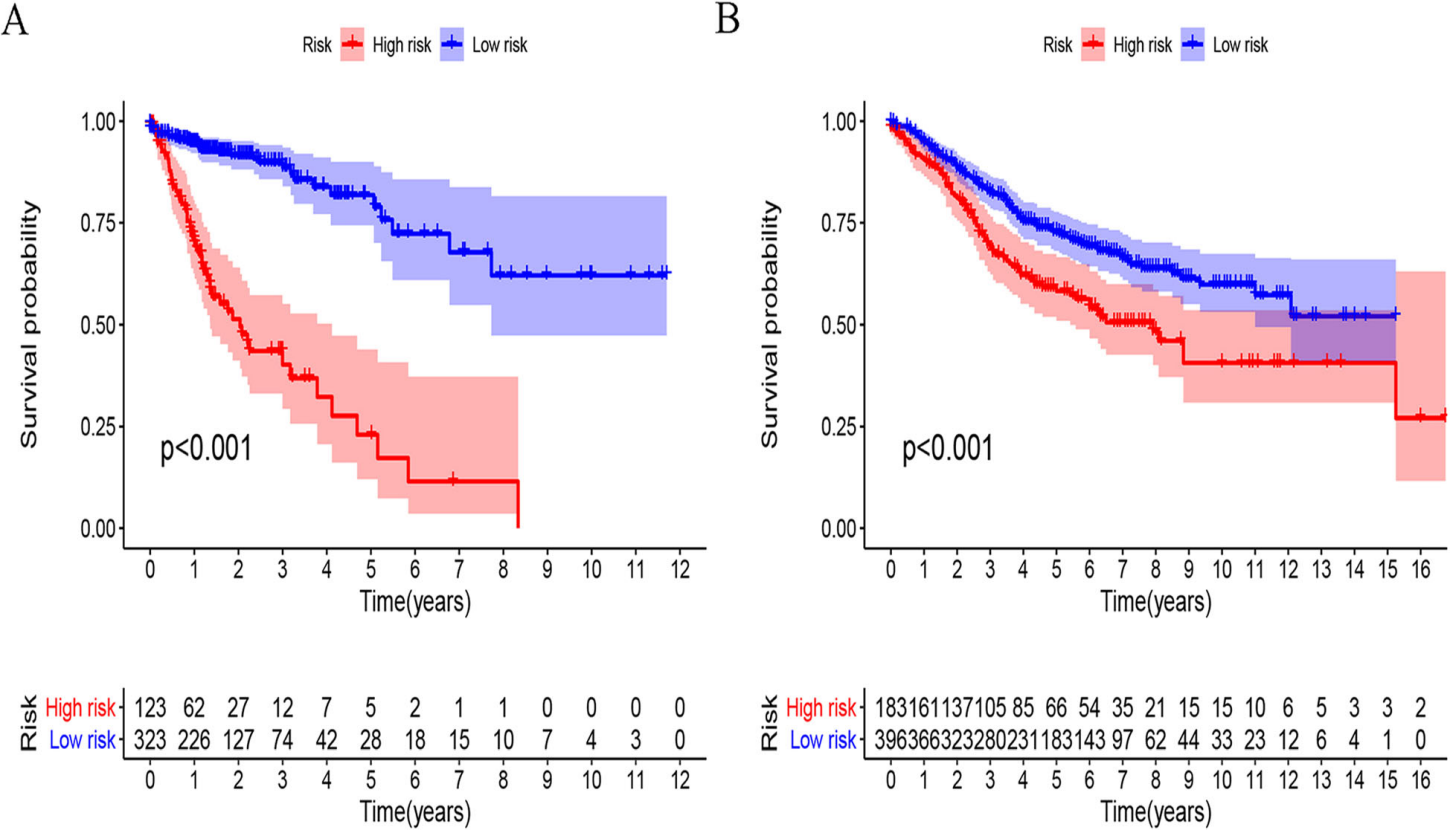
Kaplan-meier curves of OS among different risk groups. Patients were stratified by immune-related gene pairs model. OS among patients in the training (**a**) and validation cohorts(**b**). Abbreviation: OS, overall survival

**Fig. 3:**
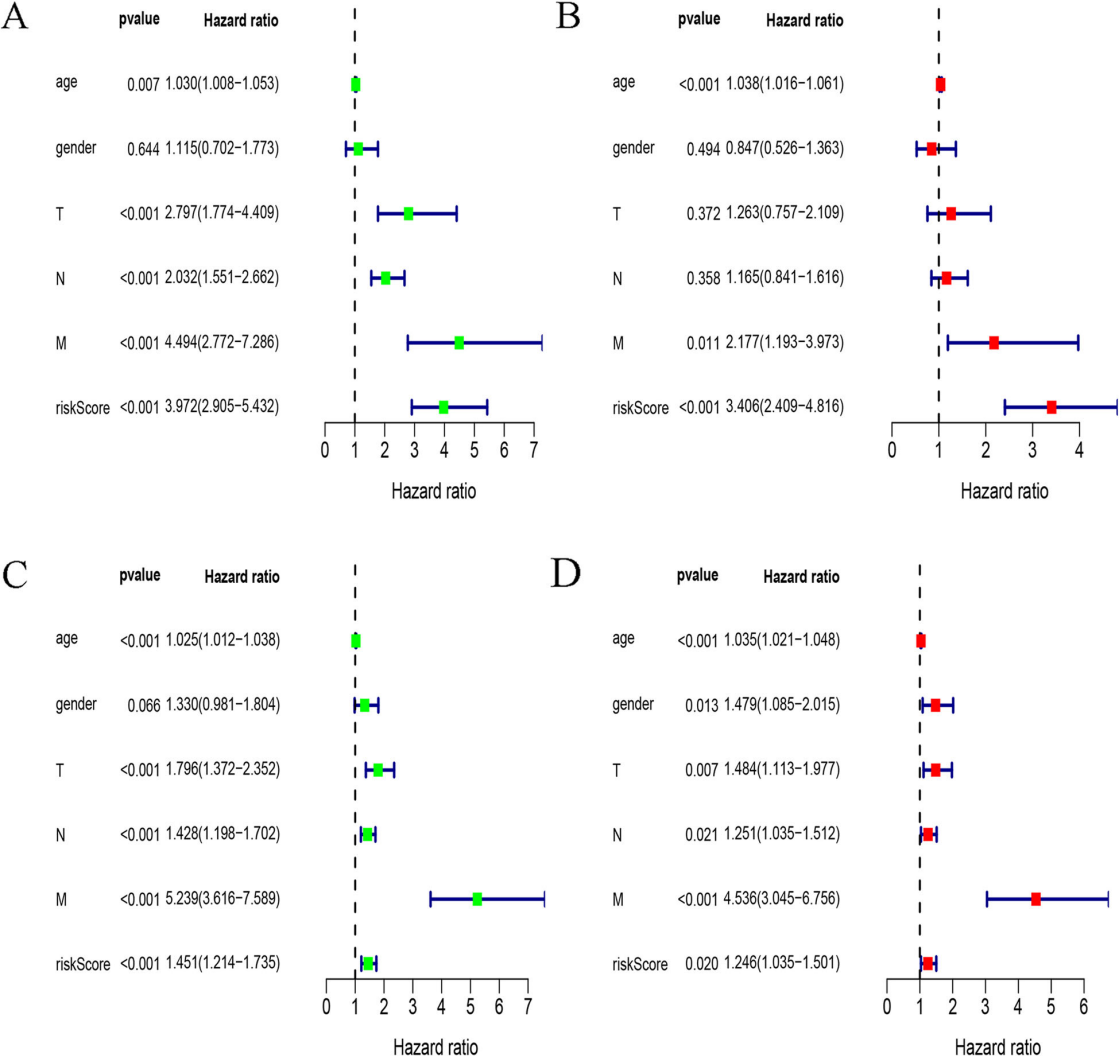
Univariate and multivariate analyses of prognostic factors in the training and validation cohort. **a** and **c** represent the univariate analysis of training cohort and validation cohort, respectively. **b** and **d** represent the multivariate analyses of the training cohort and the validation cohort, respectively

The TCGA transcriptome data were used as a training cohort. From the list of immune-related genes (IRGs) obtained by IMMPORT, the genes in the transcriptome data were searched in turn, and 1647 IRGs were identified. To ensure relatively high variation in the genes of the two platforms, we retained 325 IRGs with a median absolute deviation (MAD) > 0.5. In total, 40,375 pairs were deleted with a ratio of 0 and 1 less than 20%. Next, 12,275 immune-related gene pairs (IRGPs) were built based on these 325 IRGs. After univariate Cox regression analysis of these IRGPs in the training group, 28 potential prognostic IRGPs remained. Using Lasso Cox proportional hazards regression to define the model on the training set, 17 IRGPs were retained to form the final prognostic risk model. These IRGPs comprised 26 unique IRGs, most of which are antibiotics, cytokine receptors and cytokine-related molecules (Table 1). Next, the risk score for each patient in the TCGA dataset was calculated based on the model. Finally, we used a time-dependent ROC curve analysis to classify patients into high- or low-immune risk groups. The optimal cutoff value for the risk score was set to − 0.576 (Fig. 1). This value successfully stratified the patients in the training cohort into high- and low-risk groups. In other words, the overall survival (OS) of the low-risk group was significantly higher than that of the high-risk group (Fig. 2a). We further performed univariate and multivariate Cox proportional hazards analyses to test whether the IRGP model predicted survival independently of other prognostic factors in the TCGA cohort. Among these analyses, the risk score of the model can be used as an independent prognostic factor (Fig. 3a, b).

**Table 1:** List of immune-related genes for constructing prognostic models

IRG1	Category	IRG2	Category	Coefficient
CXCL14	Cytokines	BST2	Antimicrobials	−0.32
RBP7	Antimicrobials	PTGS2	Antimicrobials	0.26
RBP7	Antimicrobials	ARG2	Antimicrobials	0.23
APOD	Antimicrobials	IL17RB	Cytokine_Receptors	0.05
C5AR1	Chemokine_Receptors	NR3C2	Cytokine_Receptors	0.18
IL10RA	Cytokine_Receptors	TNFRSF11A	Cytokine_Receptors	0.12
STC2	Cytokines	HNF4G	Cytokine_Receptors	0.29
RBP1	Antimicrobials	STC2	Cytokines	−0.63
GNAI1	Antimicrobials	GRP	Cytokines	−0.24
CCL4	Antimicrobials	INHBB	Cytokines	−0.19
ABCC4	Antimicrobials	GRP	Cytokines	−0.28
ARG2	Antimicrobials	GRP	Cytokines	−0.37
CCR7	Antimicrobials	INHBB	Cytokines	−0.31
CD86	Antimicrobials	IL7	Cytokines	0.28
INHBB	Cytokines	PDGFC	Cytokines	0.50
TNFRSF11A	Cytokine_Receptors	LCK	NaturalKiller_Cell_Cytotoxicity	−0.34
RORC	Cytokine_Receptors	PRKCQ	TCRsignalingPathway	−0.36

*Abbreviation*: *IRG* immune-related gene

### Verification of the feasibility of the IRGP model to predict survival

To determine whether the model had consistent prognostic value in different risk groups, we applied the model to GSE39582 and GSE17538 as external validation. The patients in the verification cohort were divided into two groups according to the risk score. The OS of subgroups in the low-risk group increased significantly (Fig. 2b, Figure S1A). After performing univariate and multivariate Cox proportional hazards analyses in the validation group, we found that the results were similar to those of the training group, and the high risk score of this model suggests a poor prognostic factor (Fig. 3c, d, Figure S1B and Figure S1C).

### Immune cell infiltration in different risk groups

**Fig. 4:**
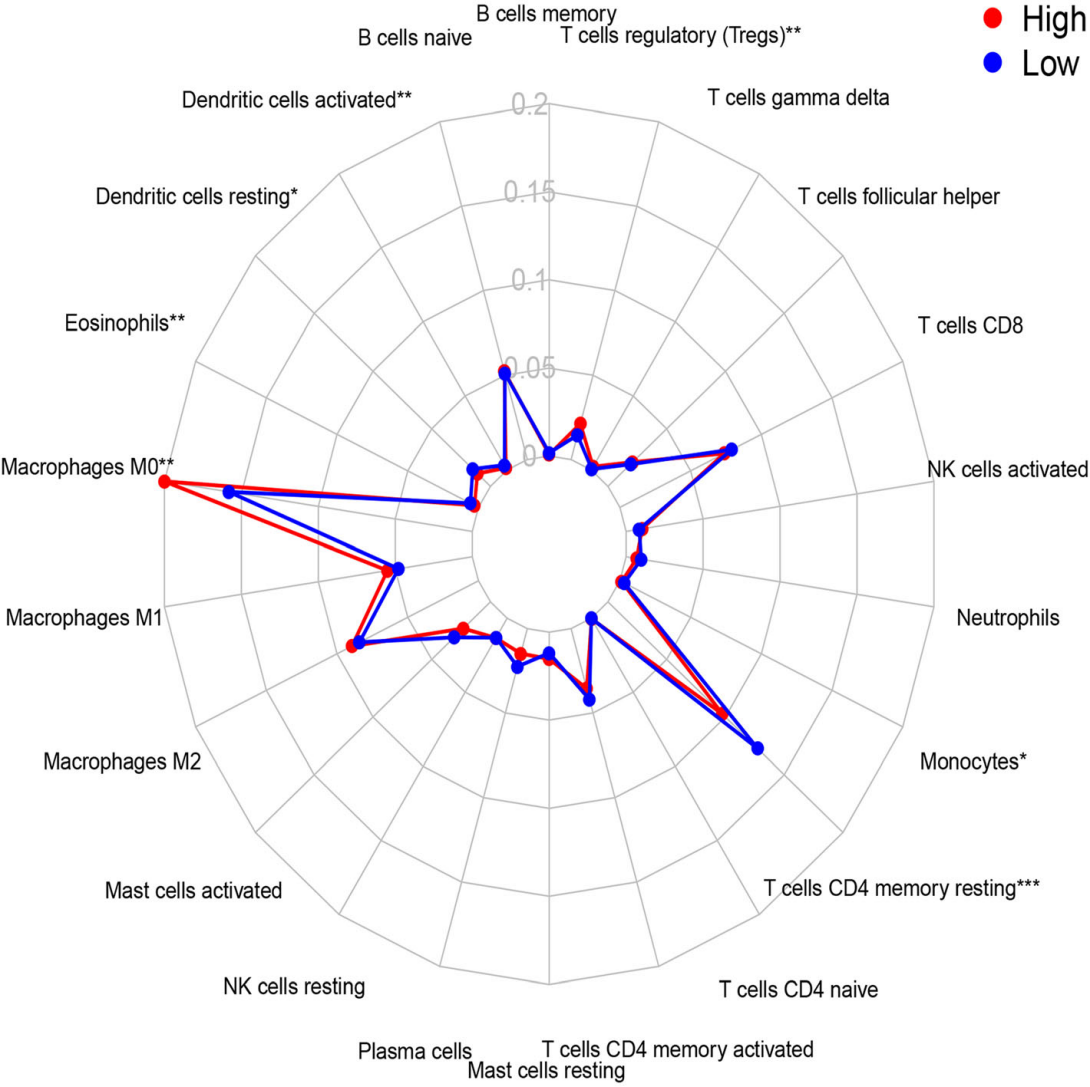
Summary of the 22 immune cells’ abundance estimated by CIBERSORT for different risk groups. *P*-values are based on t-test(*P < 0.05, **P < 0.01, ***P < 0.001)

**Fig. 5:**
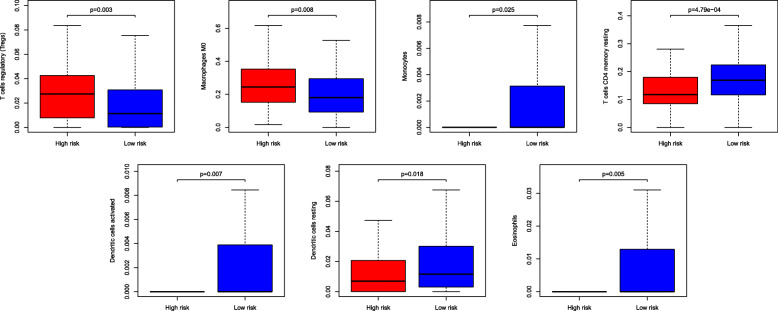
The abundance distribution of specific immune cells’ within different risk groups. T cells regulatory and Macrophage M0 were significantly highly expressed in the high-risk group, while the rest were significantly higher in the low-risk group

Previous studies have revealed that tumor-infiltrating immune cells are related to prognosis [35]. To determine the infiltration of specific tumor immune cell subsets, we used CIBERSORT to estimate the relative proportion of 22 different immune cells per patient in different risk groups. Three radar charts depict a comparative summary of various immune cells in these two risk groups (Fig. 4, Figure S2 and Figure S6). In the training cohort, we found that activated dendritic cells, resting dendritic cells, eosinophils, M0 macrophages, monocytes, resting CD4 memory T cells and regulatory T cells (Tregs) were enriched in different risk groups. Among them, regulatory T cells (Tregs) and M0 macrophages were significantly and highly expressed in the high-risk group, and the rest were highly expressed in the low-risk group (Fig. 5). The high-risk group of GSE39582 highly expressed M0 macrophages, M1 macrophages, monocytes, neutrophils, CD8 T cells and follicular helper T cells (Figure S3). The high-risk population in GSE17538 also highly expressed monocytes and Tregs (Figure S7).

### Functional evaluation of the IRGP model

**Fig. 6:**
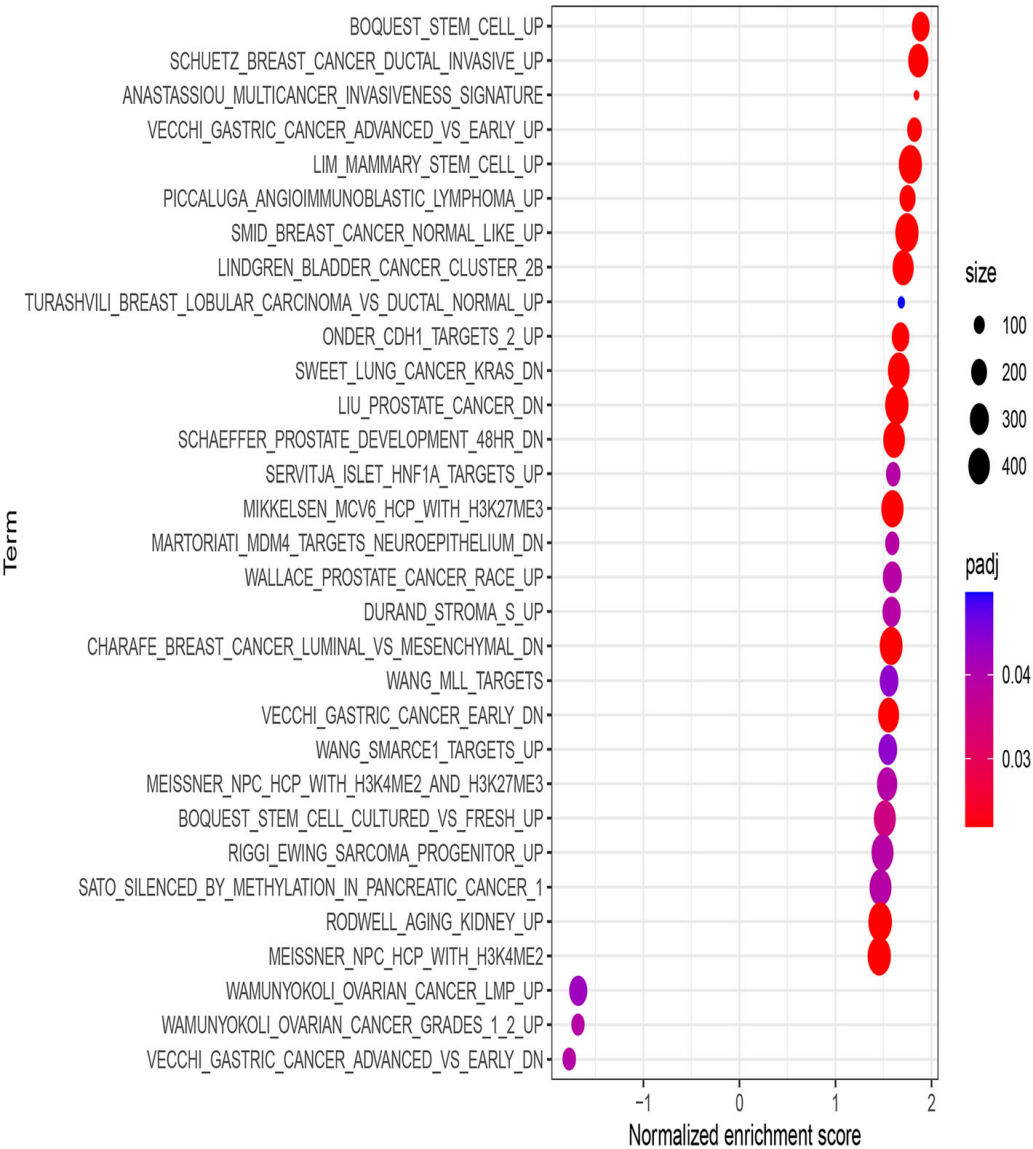
The expression characteristics of genetic perturbations significantly changed by the IRGPs model. A number of these gene sets come in pairs: xxx_UP (and xxx_DN) gene sets representing genes induced (and repressed) by the perturbation

**Fig. 7:**
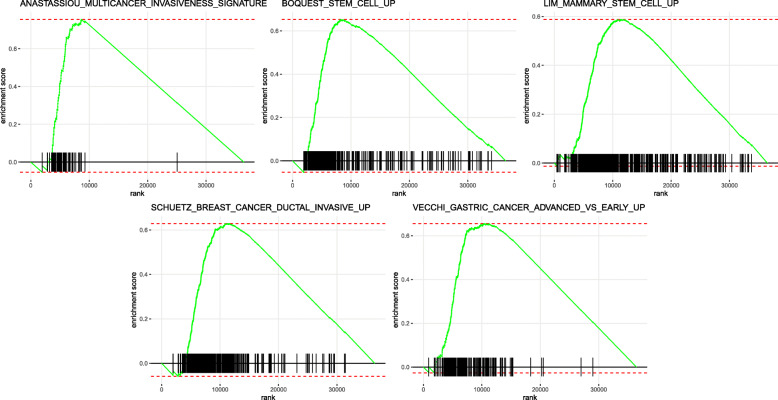
Gene Set Enrichment Analysis (GSEA). Gene set enrichment analysis confirmed that multiple tumor progression and stem cell growth-related pathways in high-risk groups were up-regulated

To investigate the expression signatures of genetic perturbations that were significantly altered by the IRGP model, GSEA was performed in the high-risk and low-risk groups in the TCGA cohort. The bubble chart revealed that genes in the high-risk populations were enriched in stem cells and various advanced tumors (Fig. 6). The top five genetic perturbations in the high-risk group were enriched stem cells, increased breast cancer ductal invasion, a multicancer invasiveness signature, increased advanced vs early-stage gastric cancer and enriched mammary stem cells (Fig. 7). We also obtained similar results when performing the above analysis on GSE39582 and GSE17538 (Figure S4, Figure S8). The high-risk group genes in GSE39582 were significantly enriched in breast cancer ductal invasion and stem cells (Figure S5). The GSEA results obtained in GSE17538 also showed that the high-risk group genes are enriched in tumor cell growth and invasion (Figure S9).

## Discussion

Colon cancer is the most common type of gastrointestinal cancer and has high morbidity and mortality. Approximately 95% of colon cancer is colon adenocarcinoma (COAD). In recent years, immunotherapy has been a hotspot in the research of major tumor types. In the COAD field, studies on the high-level microsatellite instability (MSI-H) population have been performed successively since 2015. The Keynote 016, Keynote 164, Checkmate 142, and NICHE clinical trial results all indicate the extraordinary efficacy of immunotherapy [36–39]. Patients with MSI-H have a better prognosis than those with microsatellite stability (MSS). However, the MSI-H population accounts for only approximately 10% of COAD. Most patients still face the dilemma of not having an effective prognostic indicator. Thus, the determination of new prognostic biomarkers is urgent to predict the survival of colon adenocarcinoma patients.

To obtain the robustness of the prognosis prediction in this study, we adopted a method for data analysis without considering the technical deviation of different platforms. The newly established prognostic model is based on the ranking and pairing comparison of relative gene expression values; thus, data preprocessing, such as scaling and normalization, is not required. This method has reliable results in many studies [40, 41].

In this study, we identified an immune-related gene pair model to predict the overall survival for colon adenocarcinoma. The prognostic model comprises 17 immune-related gene pairs containing 26 unique immune-related genes. Most genes in this immune model are cytokine receptors and cytokines, which play a vital role in the adaptive immune response. Among these IRGs, no evidence supports that the overexpression of IL17RB can enhance the invasion and metastasis of thyroid cancer cells [42]. STC2 overexpression is associated with a poor prognosis in patients with nasopharyngeal carcinoma (NPC) and can be used as a predictor of NPC responses to radiation [43]. The increase in IL-7 in colorectal cancer (CRC) is related to metastatic disease and tumor location [44]. Decreased CXCL14 expression indicates a poor prognosis and causes metastasis in colon cancer [45]. GRP signaling alters the invasion of colon cancer through heterochromatin protein 1^Hsβ^ and can improve the prognosis of patients with colon cancer [46]. Moreover, regulatory T cells (Tregs) and M0 macrophages are related to the poor clinical prognosis of many patients with cancer [47, 48]. Dendritic cells are associated with cancer immunity and a favorable prognosis [49]. At the same time, the immune cell types M0 macrophages, M1 macrophages, monocytes, neutrophils, CD8 T cells and follicular helper T cells in the high-risk group of GSE39582 are all related to tumor progression and poor prognosis [50–53]. These findings are consistent with our results. In this study, we also found that several expression characteristics of genetic perturbations, such as increased stem cells, increased breast cancer ductal invasion, a multicancer invasiveness signature, increased advanced vs early gastric cancer and increased mammary stem cells, were related to the IRGP model. These results were verified by corresponding experiments [54–58], confirming their importance in tumor development and cell growth. These findings indicate that the IRGP model may play an essential role in tumor invasiveness and progression in COAD.

The difference between this study and previously published studies [59] is that the IRGP model was established based on the TCGA database. Second, our strategy to establish a prognostic model was different. To screen out immune-related gene pairs that are significantly related to OS in patients with colon cancer, we used univariate Cox regression analysis before determining the final model using Lasso regression analysis. Finally, we conducted GSEA in the training and validation cohorts to further analyze the specific differences between the high- and low-risk groups. We found that the high-risk group genes were significantly enriched in tumor cell invasion and growth.

Similar to all RNA-seq and microarray analyses, our study had limitations. First, the training dataset to build the immune model was obtained from a retrospective study, which included fresh frozen samples; the stability and efficiency of formalin-fixed and paraffin-embedded (FFPE) samples remain questionable. Therefore, it may be necessary to add more datasets with different sample attributes for more extensive verification. Second, because the prognostic model was based on TCGA and other databases, it required proficiency in bioinformatics. Additionally, the gene expression profiles produced by RNA-seq or microarray platforms require high prices and long conversion cycles. Therefore, this method is challenging to popularize in daily clinical applications.

## Conclusions

In summary, our immune-related gene pair model can provide an evaluation reference for the prognostic risk of patients with colon adenocarcinoma. The immune-related model was associated with the prognosis of patients with COAD. The tumor-infiltrating immune cells and genetic perturbations distinguished by this model in the high- and low-risk groups can further elucidate the role of our prognostic model in the development of colon adenocarcinoma. Therefore, the risk model will be a useful tool to better evaluate patients who may benefit from immunotherapy.

## Supplementary information

**Additional file 1: Figure S1.** Additional verification using GSE17538. Patients were stratified by immune-related gene pairs model(A). (B) represent the univariate analysis result and (C) represent the multivariate analyses result.

**Additional file 2: Figure S2.** Summary of the 22 immune cells’ abundance estimated by CIBERSORT for different risk groups in GSE39582. *P*-values are based on t-test(*P < 0.05, **P < 0.01, ***P < 0.001).

**Additional file 3: Figure S3.** The abundance distribution of specific immune cells’ within different risk groups in GSE39582.

**Additional file 4: Figure S4.** The expression characteristics of genetic perturbations significantly changed by the IRGPs model in GSE39582.

**Additional file 5: Figure S5.** The top 5 results of GSEA in GSE39582.

**Additional file 6: Figure S6.** Summary of the 22 immune cells’ abundance estimated by CIBERSORT for different risk groups in GSE17538. *P*-values are based on t-test(*P < 0.05, **P < 0.01, ***P < 0.001).

**Additional file 7: Figure S7.** The abundance distribution of specific immune cells’ within different risk groups in GSE17538.

**Additional file 8: Figure S8.** The expression characteristics of genetic perturbations significantly changed by the IRGPs model in GSE17538.

**Additional file 9: Figure S9.** The top 5 results of GSEA in GSE17538.

## Data Availability

The datasets analyzed in this study can be found in the Gene Expression Omnibus (https://www.ncbi.nlm.nih.gov/geo/) and TCGA (https://portal.gdc.cancer.gov/).

## Abbreviations

COAD: Colon adenocarcinoma
CRC: Colorectal cancer
FFPE: Formalin-fixed and paraffin-embedded
GARP: Glycoprotein-A repetitions predominant
GEPs: Gene expression profiles
GSEA: Gene set enrichment analysis
hunman.gtf: Human general transfer format
IRGPs: Immune-related gene pairs
IRGs: Immune-related genes
MAD: Median absolute deviation
MSI-H: High-level microsatellite instability
MSS: Microsatellite stability
NPC: Nasopharyngeal carcinoma
OS: Overall survival
ROC: Receiver operating characteristic
Tregs: Regulatory T cells

## References

[1] Bray F, Ferlay J, Soerjomataram I, Siegel RL, Torre LA, Jemal A (2018). Global cancer statistics 2018: GLOBOCAN estimates of incidence and mortality worldwide for 36 cancers in 185 countries. CA Cancer J Clin.

[2] Siegel RL, Miller KD, Jemal A (2019). Cancer statistics, 2019. CA Cancer J Clin.

[3] Center MM, Jemal A, Smith RA, Ward E (2009). Worldwide variations in colorectal cancer. CA Cancer J Clin.

[4] Bertelsen CA, Larsen HM, Neuenschwander AU, Laurberg S, Kristensen B, Emmertsen KJ (2018). Long-term functional outcome after right-sided complete Mesocolic excision compared with conventional Colon Cancer surgery: a population-based questionnaire study. Dis Colon Rectum.

[5] Brungs D, Aghmesheh M, de Souza P, Carolan M, Clingan P, Rose J (2018). Safety and efficacy of Oxaliplatin doublet adjuvant chemotherapy in elderly patients with stage III Colon Cancer. Clin Colorectal Cancer.

[6] Grothey A, Sobrero AF, Shields AF, Yoshino T, Paul J, Taieb J (2018). Duration of adjuvant chemotherapy for stage III Colon Cancer. N Engl J Med.

[7] Guo M, Xu E, Ai D (2019). Inferring bacterial infiltration in primary colorectal tumors from host whole genome sequencing data. Front Genet.

[8] Okugawa Y, Grady WM, Goel A (2015). Epigenetic alterations in colorectal Cancer: emerging biomarkers. Gastroenterology..

[9] Allen J, Sears CL (2019). Impact of the gut microbiome on the genome and epigenome of colon epithelial cells: contributions to colorectal cancer development. Genome Med.

[10] Vasaikar S, Huang C, Wang X, Petyuk VA, Savage SR, Wen B (2019). Proteogenomic analysis of human Colon Cancer reveals new therapeutic opportunities. Cell..

[11] Choi MR, Gwak M, Yoo NJ, Lee SH (2015). Regional Bias of Intratumoral genetic heterogeneity of apoptosis-related genes BAX, APAF1, and FLASH in Colon cancers with high microsatellite instability. Dig Dis Sci.

[12] Sugai T, Eizuka M, Takahashi Y, Fukagawa T, Habano W, Yamamoto E (2017). Molecular subtypes of colorectal cancers determined by PCR-based analysis. Cancer Sci.

[13] Mamlouk S, Childs LH, Aust D, Heim D, Melching F, Oliveira C (2017). DNA copy number changes define spatial patterns of heterogeneity in colorectal cancer. Nat Commun.

[14] Hsu YL, Lin CC, Jiang JK, Lin HH, Lan YT, Wang HS (2019). Clinicopathological and molecular differences in colorectal cancer according to location. Int J Biol Markers.

[15] Liu T, Li C, Jin L, Li C, Wang L (2019). The Prognostic Value of m6A RNA Methylation Regulators in Colon Adenocarcinoma. Med Sci Monit.

[16] Missiaglia E, Jacobs B, D'Ario G, Di Narzo AF, Soneson C, Budinska E (2014). Distal and proximal colon cancers differ in terms of molecular, pathological, and clinical features. Ann Oncol.

[17] Patel SA, Minn AJ (2018). Combination Cancer therapy with immune checkpoint blockade: mechanisms and strategies. Immunity..

[18] Woo SR, Corrales L, Gajewski TF (2015). Innate immune recognition of cancer. Annu Rev Immunol.

[19] Gentles AJ, Newman AM, Liu CL, Bratman SV, Feng W, Kim D (2015). The prognostic landscape of genes and infiltrating immune cells across human cancers. Nat Med.

[20] Salem M, Wallace C, Velegraki M, Li A, Ansa-Addo E, Metelli A (2019). GARP dampens Cancer immunity by sustaining function and accumulation of regulatory T cells in the Colon. Cancer Res.

[21] Heinaniemi M, Nykter M, Kramer R, Wienecke-Baldacchino A, Sinkkonen L, Zhou JX (2013). Gene-pair expression signatures reveal lineage control. Nat Methods.

[22] Li B, Cui Y, Diehn M, Li R (2017). Development and validation of an individualized immune prognostic signature in early-stage nonsquamous non-small cell lung Cancer. JAMA Oncol.

[23] Wei HT, Guo EN, Liao XW, Chen LS, Wang JL, Ni M (2018). Genomescale analysis to identify potential prognostic microRNA biomarkers for predicting overall survival in patients with colon adenocarcinoma. Oncol Rep.

[24] Marisa L, de Reynies A, Duval A, Selves J, Gaub MP, Vescovo L (2013). Gene expression classification of colon cancer into molecular subtypes: characterization, validation, and prognostic value. PLoS Med.

[25] Smith JJ, Deane NG, Wu F, Merchant NB, Zhang B, Jiang A (2010). Experimentally derived metastasis gene expression profile predicts recurrence and death in patients with colon cancer. Gastroenterology..

[26] Cunningham F, Achuthan P, Akanni W, Allen J, Amode MR, Armean IM (2019). Ensembl 2019. Nucleic Acids Res.

[27] Liu W, Islamaj Dogan R, Kwon D, Marques H, Rinaldi F, Wilbur WJ, et al. BioC implementations in Go, Perl, Python and Ruby. Database (Oxford). 2014;2014. 10.1093/database/bau059. 10.1093/database/bau059 PMC4067548 24961236

[28] Bhattacharya S, Dunn P, Thomas CG, Smith B, Schaefer H, Chen J (2018). ImmPort, toward repurposing of open access immunological assay data for translational and clinical research. Sci Data.

[29] Huber W, Carey VJ, Gentleman R, Anders S, Carlson M, Carvalho BS (2015). Orchestrating high-throughput genomic analysis with bioconductor. Nat Methods.

[30] Guinney J, Dienstmann R, Wang X, de Reynies A, Schlicker A, Soneson C (2015). The consensus molecular subtypes of colorectal cancer. Nat Med.

[31] Wu M, Li X, Zhang T, Liu Z, Zhao Y (2019). Identification of a nine-gene signature and establishment of a prognostic Nomogram predicting overall survival of pancreatic Cancer. Front Oncol.

[32] Wan B, Liu B, Huang Y, Yu G, Lv C (2019). Prognostic value of immune-related genes in clear cell renal cell carcinoma. Aging (Albany NY).

[33] Newman AM, Liu CL, Green MR, Gentles AJ, Feng W, Xu Y (2015). Robust enumeration of cell subsets from tissue expression profiles. Nat Methods.

[34] Subramanian A, Tamayo P, Mootha VK, Mukherjee S, Ebert BL, Gillette MA (2005). Gene set enrichment analysis: a knowledge-based approach for interpreting genome-wide expression profiles. Proc Natl Acad Sci U S A.

[35] Domingues P, Gonzalez-Tablas M, Otero A, Pascual D, Miranda D, Ruiz L (2016). Tumor infiltrating immune cells in gliomas and meningiomas. Brain Behav Immun.

[36] Overman MJ, McDermott R, Leach JL, Lonardi S, Lenz H-J, Morse MA (2017). Nivolumab in patients with metastatic DNA mismatch repair-deficient or microsatellite instability-high colorectal cancer (CheckMate 142): an open-label, multicentre, phase 2 study. The Lancet Oncology.

[37] Chalabi M, Fanchi LF, Dijkstra KK, Van den Berg JG, Aalbers AG, Sikorska K (2020). Neoadjuvant immunotherapy leads to pathological responses in MMR-proficient and MMR-deficient early-stage colon cancers. Nat Med.

[38] Le DT, Uram JN, Wang H, Bartlett BR, Kemberling H, Eyring AD (2015). PD-1 blockade in tumors with mismatch-repair deficiency. N Engl J Med.

[39] Le DT, Kim TW, Van Cutsem E, Geva R, Jager D, Hara H (2020). Phase II open-label study of Pembrolizumab in treatment-refractory, microsatellite instability-high/mismatch repair-deficient metastatic colorectal Cancer: KEYNOTE-164. J Clin Oncol.

[40] Eddy JA, Sung J, Geman D, Price ND (2010). Relative expression analysis for molecular cancer diagnosis and prognosis. Technol Cancer Res Treat.

[41] Popovici V, Budinska E, Tejpar S, Weinrich S, Estrella H, Hodgson G (2012). Identification of a poor-prognosis BRAF-mutant-like population of patients with colon cancer. J Clin Oncol.

[42] Ren L, Xu Y, Liu C, Wang S, Qin G (2017). IL-17RB enhances thyroid cancer cell invasion and metastasis via ERK1/2 pathway-mediated MMP-9 expression. Mol Immunol.

[43] Lin S, Guo Q, Wen J, Li C, Lin J, Cui X (2014). Survival analyses correlate stanniocalcin 2 overexpression to poor prognosis of nasopharyngeal carcinomas. J Exp Clin Cancer Res.

[44] Krzystek-Korpacka M, Zawadzki M, Neubauer K, Bednarz-Misa I, Gorska S, Wisniewski J (2017). Elevated systemic interleukin-7 in patients with colorectal cancer and individuals at high risk of cancer: association with lymph node involvement and tumor location in the right colon. Cancer Immunol Immunother.

[45] Liu J, Wang D, Zhang C, Zhang Z, Chen X, Lian J (2018). Identification of liver metastasis-associated genes in human colon carcinoma by mRNA profiling. Chin J Cancer Res.

[46] Tell R, Rivera CA, Eskra J, Taglia LN, Blunier A, Wang QT (2011). Gastrin-releasing peptide signaling alters colon cancer invasiveness via heterochromatin protein 1Hsbeta. Am J Pathol.

[47] Najafi M, Farhood B, Mortezaee K (2019). Contribution of regulatory T cells to cancer: a review. J Cell Physiol.

[48] Liu X, Wu S, Yang Y, Zhao M, Zhu G, Hou Z (2017). The prognostic landscape of tumor-infiltrating immune cell and immunomodulators in lung cancer. Biomed Pharmacother.

[49] Veglia F, Gabrilovich DI (2017). Dendritic cells in cancer: the role revisited. Curr Opin Immunol.

[50] Giese MA, Hind LE, Huttenlocher A (2019). Neutrophil plasticity in the tumor microenvironment. Blood..

[51] Olingy CE, Dinh HQ, Hedrick CC (2019). Monocyte heterogeneity and functions in cancer. J Leukoc Biol.

[52] Reading JL, Galvez-Cancino F, Swanton C, Lladser A, Peggs KS, Quezada SA (2018). The function and dysfunction of memory CD8(+) T cells in tumor immunity. Immunol Rev.

[53] Townsend W, Pasikowska M, Yallop D, Phillips EH, Patten PEM, Salisbury JR (2020). The architecture of neoplastic follicles in follicular lymphoma; analysis of the relationship between the tumor and follicular helper T cells. Haematologica..

[54] Taranger CK, Noer A, Sorensen AL, Hakelien AM, Boquest AC, Collas P (2005). Induction of dedifferentiation, genomewide transcriptional programming, and epigenetic reprogramming by extracts of carcinoma and embryonic stem cells. Mol Biol Cell.

[55] Hennigs A, Fuchs V, Sinn HP, Riedel F, Rauch G, Smetanay K (2016). Do patients after Reexcision due to involved or close margins have the same risk of local recurrence as those after one-step breast-conserving surgery?. Ann Surg Oncol.

[56] Anastassiou D, Rumjantseva V, Cheng W, Huang J, Canoll PD, Yamashiro DJ (2011). Human cancer cells express slug-based epithelial-mesenchymal transition gene expression signature obtained in vivo. BMC Cancer.

[57] Vecchi M, Nuciforo P, Romagnoli S, Confalonieri S, Pellegrini C, Serio G (2007). Gene expression analysis of early and advanced gastric cancers. Oncogene..

[58] Fu NY, Pal B, Chen Y, Jackling FC, Milevskiy M, Vaillant F (2018). Foxp1 is indispensable for ductal morphogenesis and controls the exit of mammary stem cells from quiescence. Dev Cell.

[59] Wu J, Zhao Y, Zhang J, Wu Q, Wang W (2019). Development and validation of an immune-related gene pairs signature in colorectal cancer. Oncoimmunology..

