# Supplementary figures and images for: Establishment of an immune-related gene pair model to predict colon adenocarcinoma prognosis
Source: BMC Cancer. 2020 Nov 9;20:1071. doi: 10.1186/s12885-020-07532-7 (PMC7654612; doi:10.1186/s12885-020-07532-7)

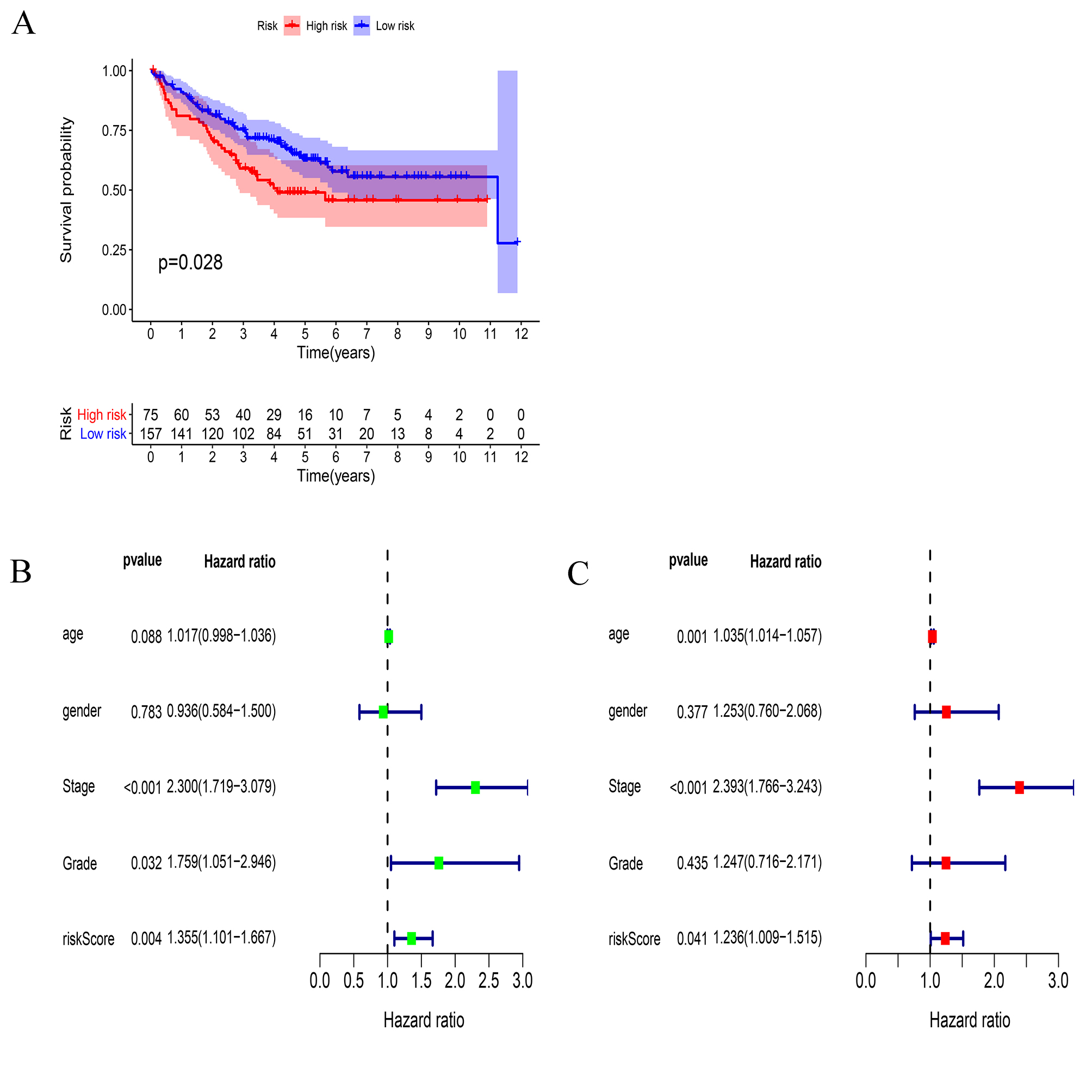

Supplement: Supplementary file 1 — Additional file 1: Figure S1. Additional verification using GSE17538. Patients were stratified by immune-related gene pairs model(A). (B) represent the univariate analysis result and (C) represent the multivariate analyses result. [file 12885_2020_7532_MOESM1_ESM.jpg]

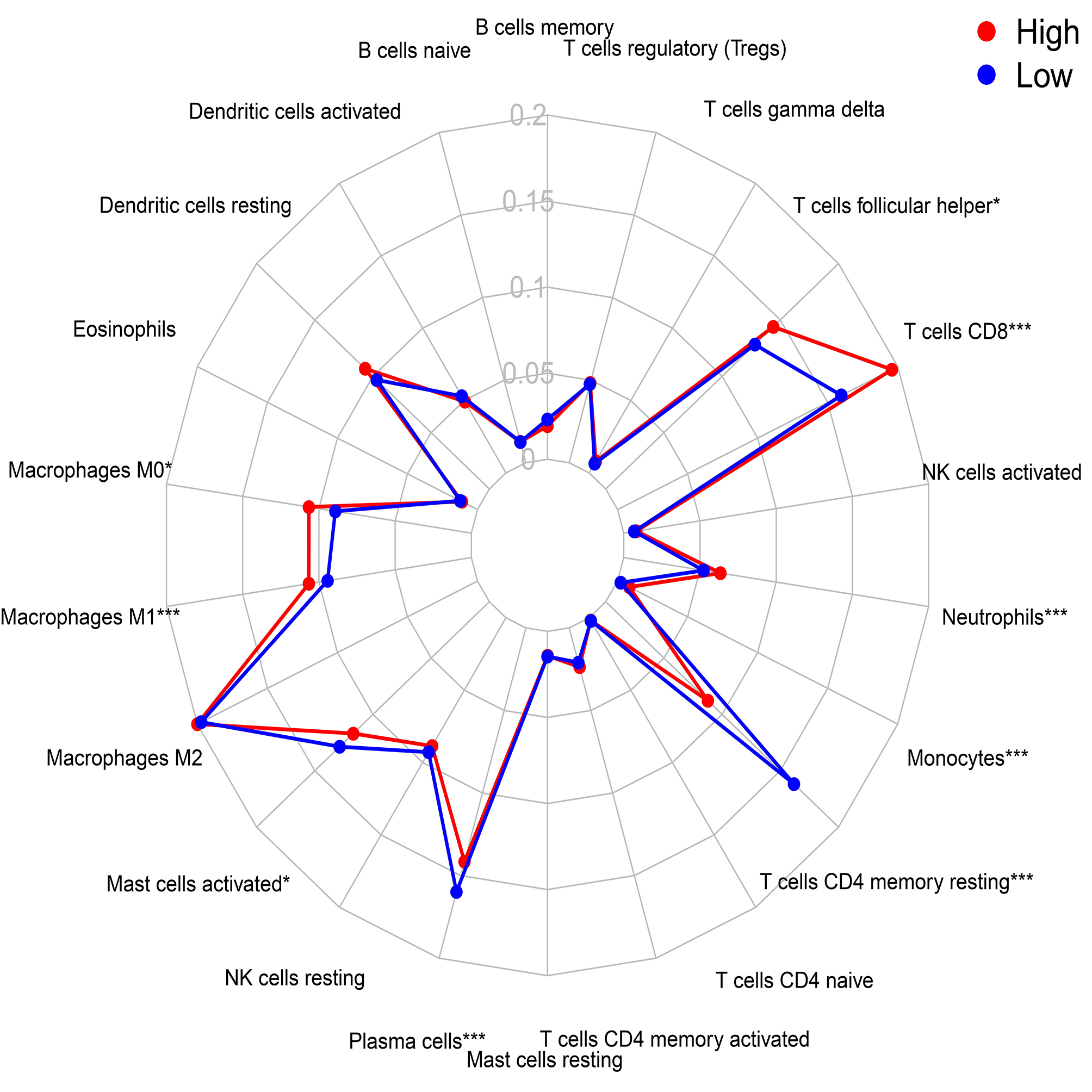

Supplement: Supplementary file 2 — Additional file 2: Figure S2. Summary of the 22 immune cells’ abundance estimated by CIBERSORT for different risk groups in GSE39582. P-values are based on t-test(*P < 0.05, **P < 0.01, ***P < 0.001). [file 12885_2020_7532_MOESM2_ESM.jpg]

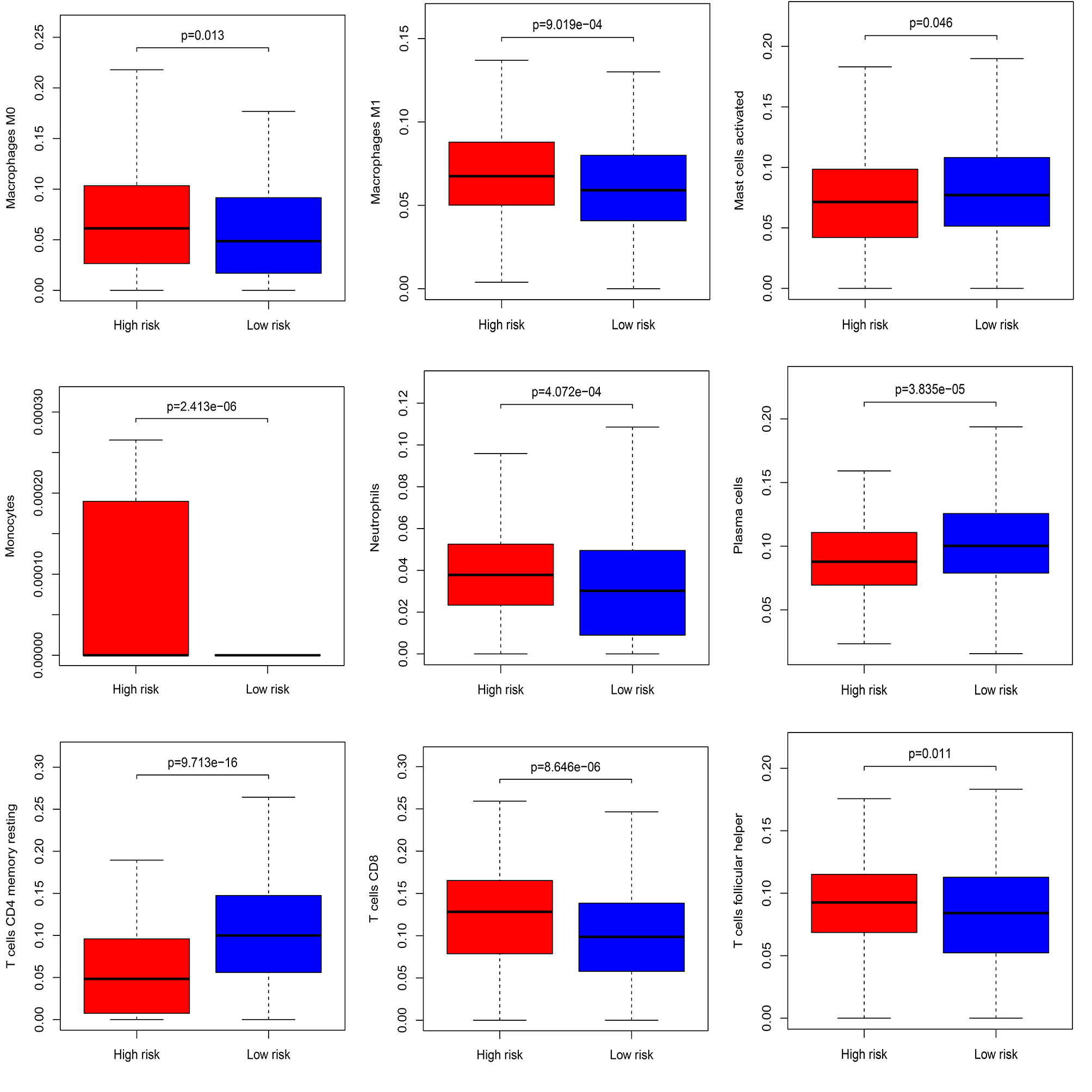

Supplement: Supplementary file 3 — Additional file 3: Figure S3. The abundance distribution of specific immune cells’ within different risk groups in GSE39582. [file 12885_2020_7532_MOESM3_ESM.jpg]

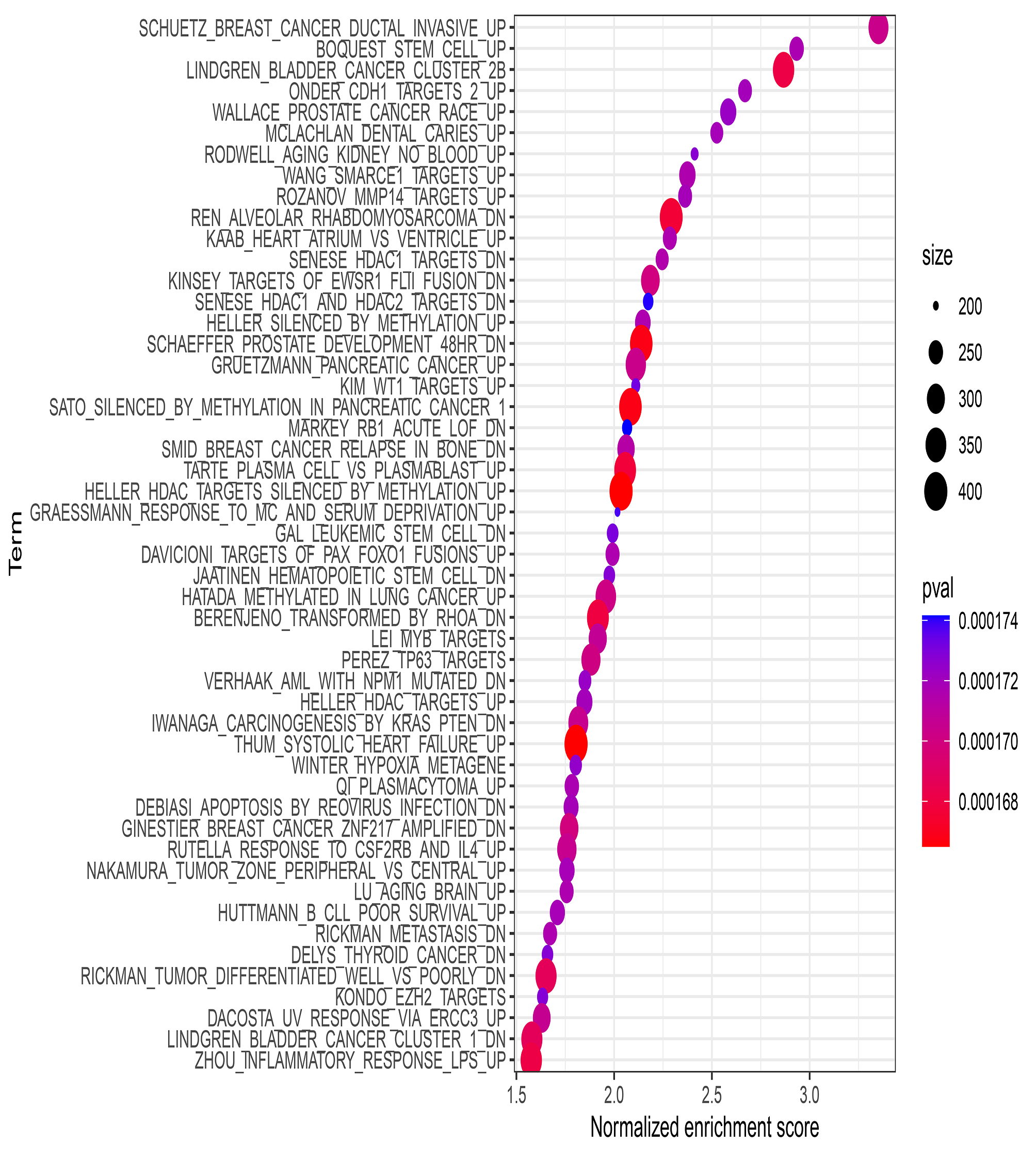

Supplement: Supplementary file 4 — Additional file 4: Figure S4. The expression characteristics of genetic perturbations significantly changed by the IRGPs model in GSE39582. [file 12885_2020_7532_MOESM4_ESM.jpg]

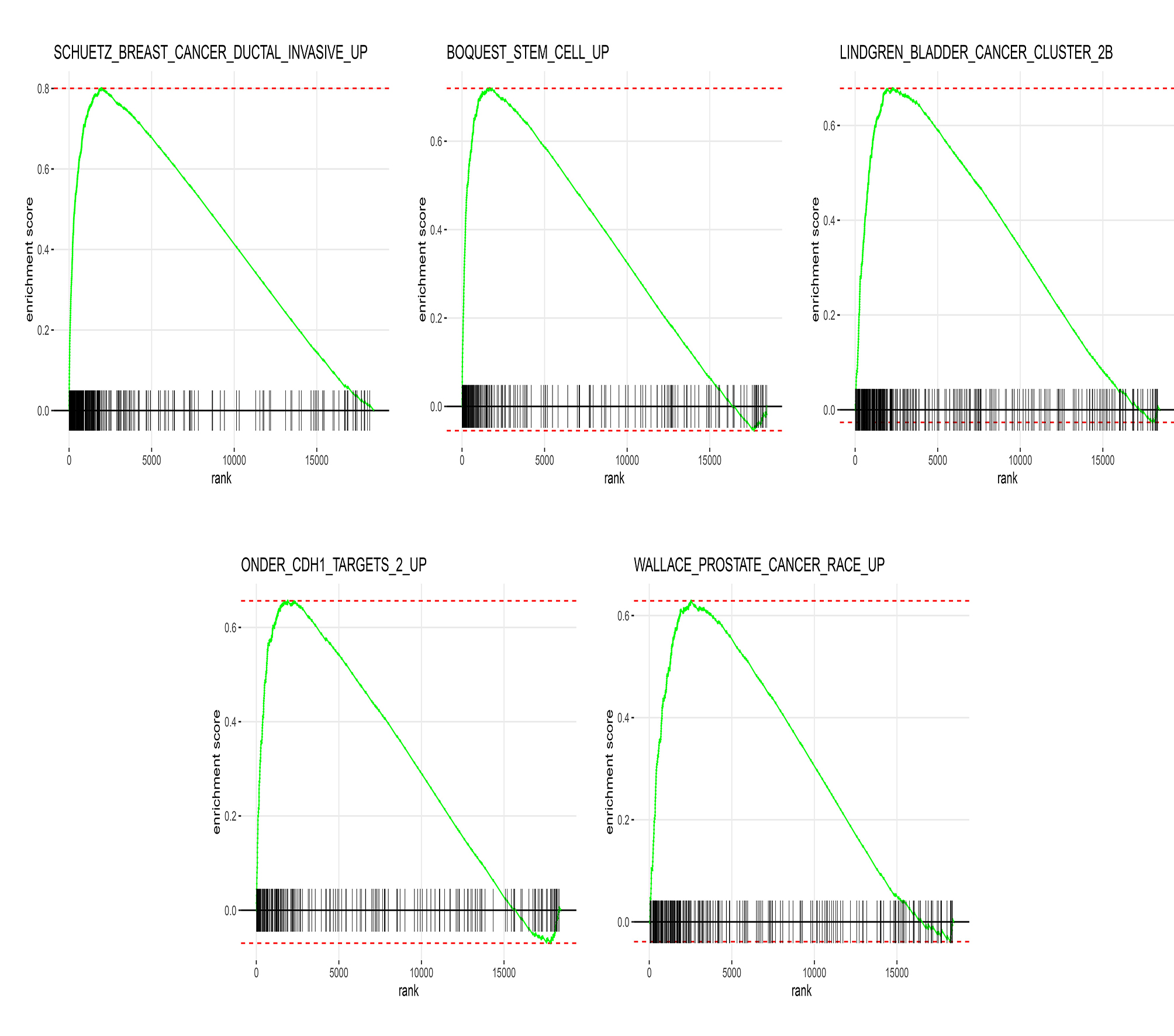

Supplement: Supplementary file 5 — Additional file 5: Figure S5. The top 5 results of GSEA in GSE39582. [file 12885_2020_7532_MOESM5_ESM.jpg]

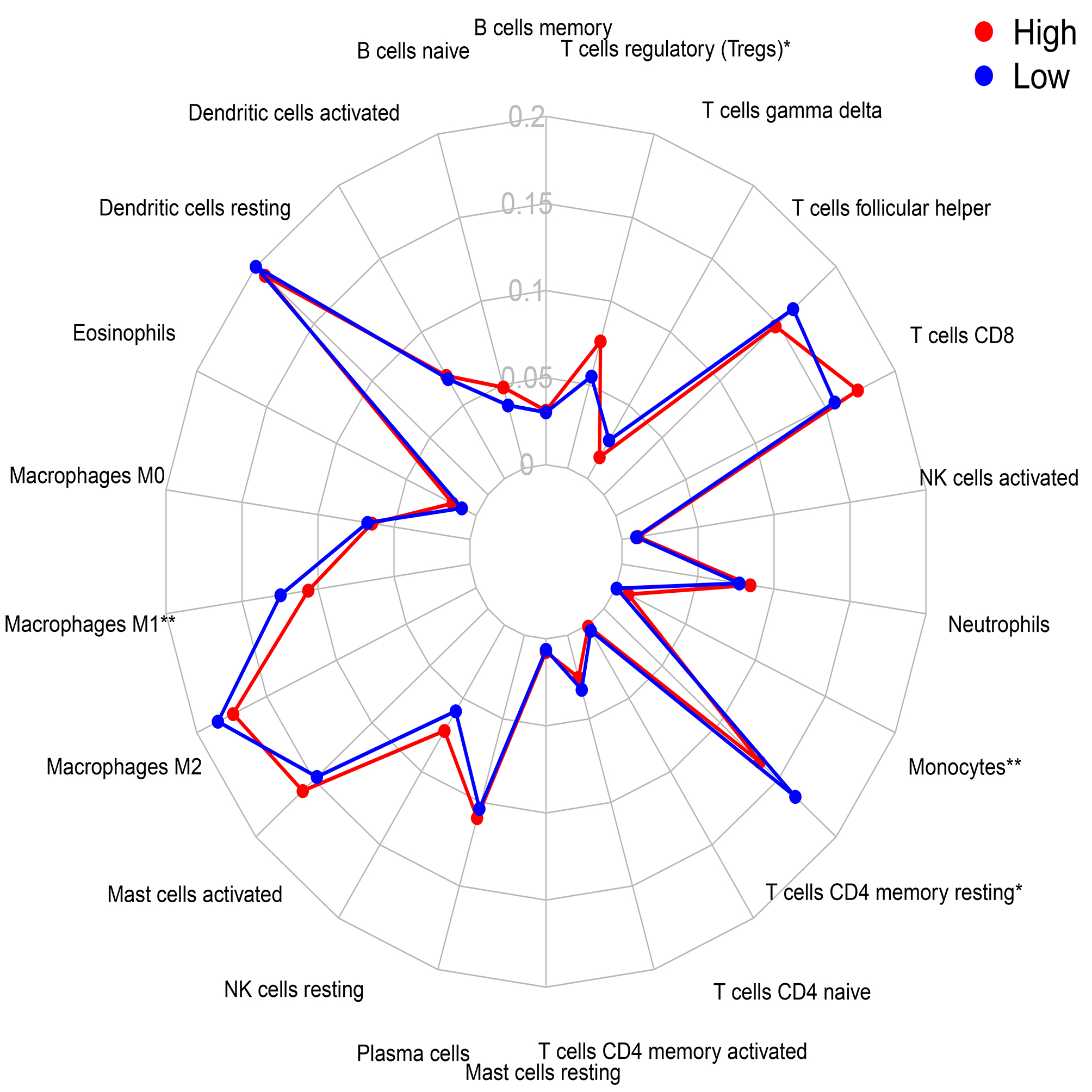

Supplement: Supplementary file 6 — Additional file 6: Figure S6. Summary of the 22 immune cells’ abundance estimated by CIBERSORT for different risk groups in GSE17538. P-values are based on t-test(*P < 0.05, **P < 0.01, ***P < 0.001). [file 12885_2020_7532_MOESM6_ESM.jpg]

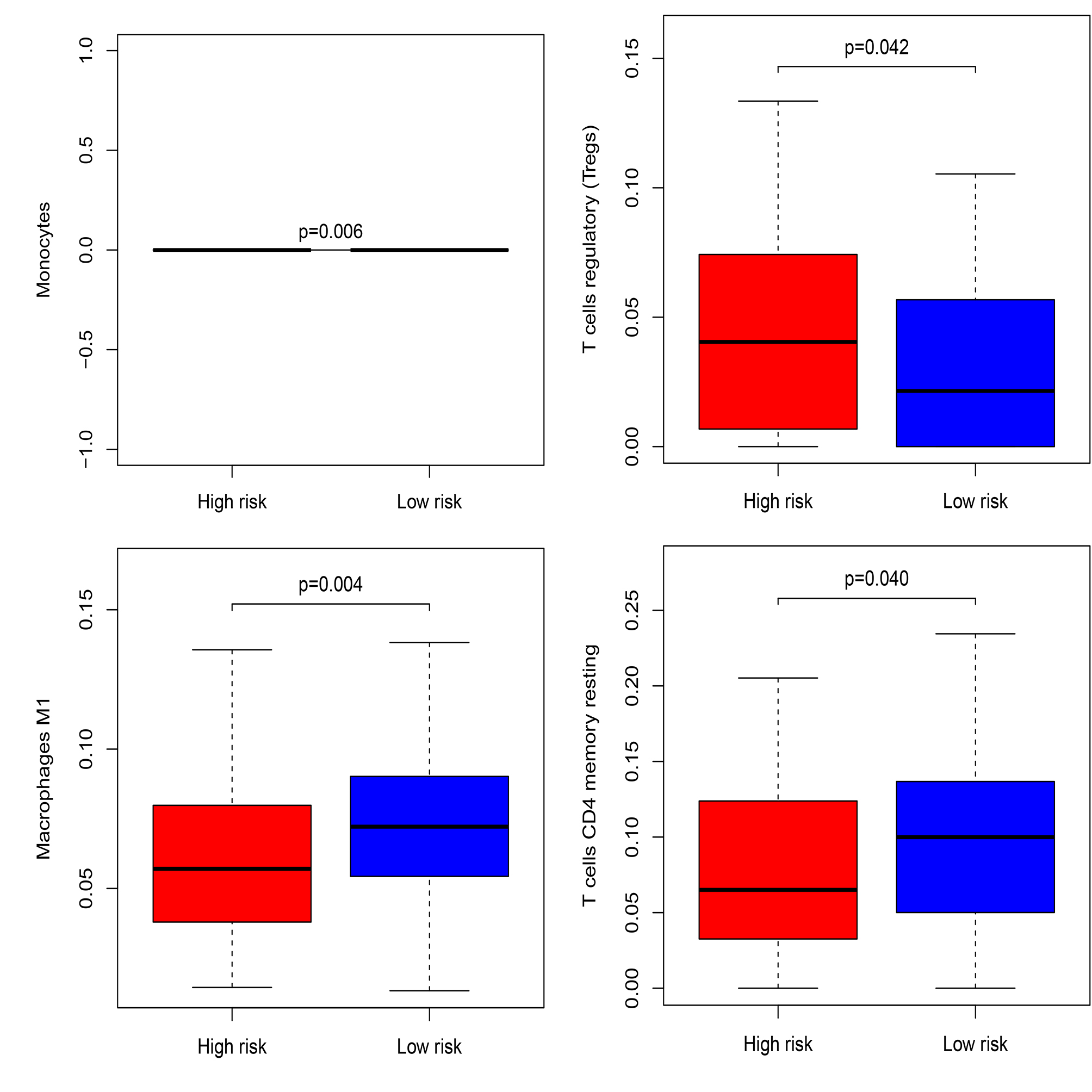

Supplement: Supplementary file 7 — Additional file 7: Figure S7. The abundance distribution of specific immune cells’ within different risk groups in GSE17538. [file 12885_2020_7532_MOESM7_ESM.jpg]

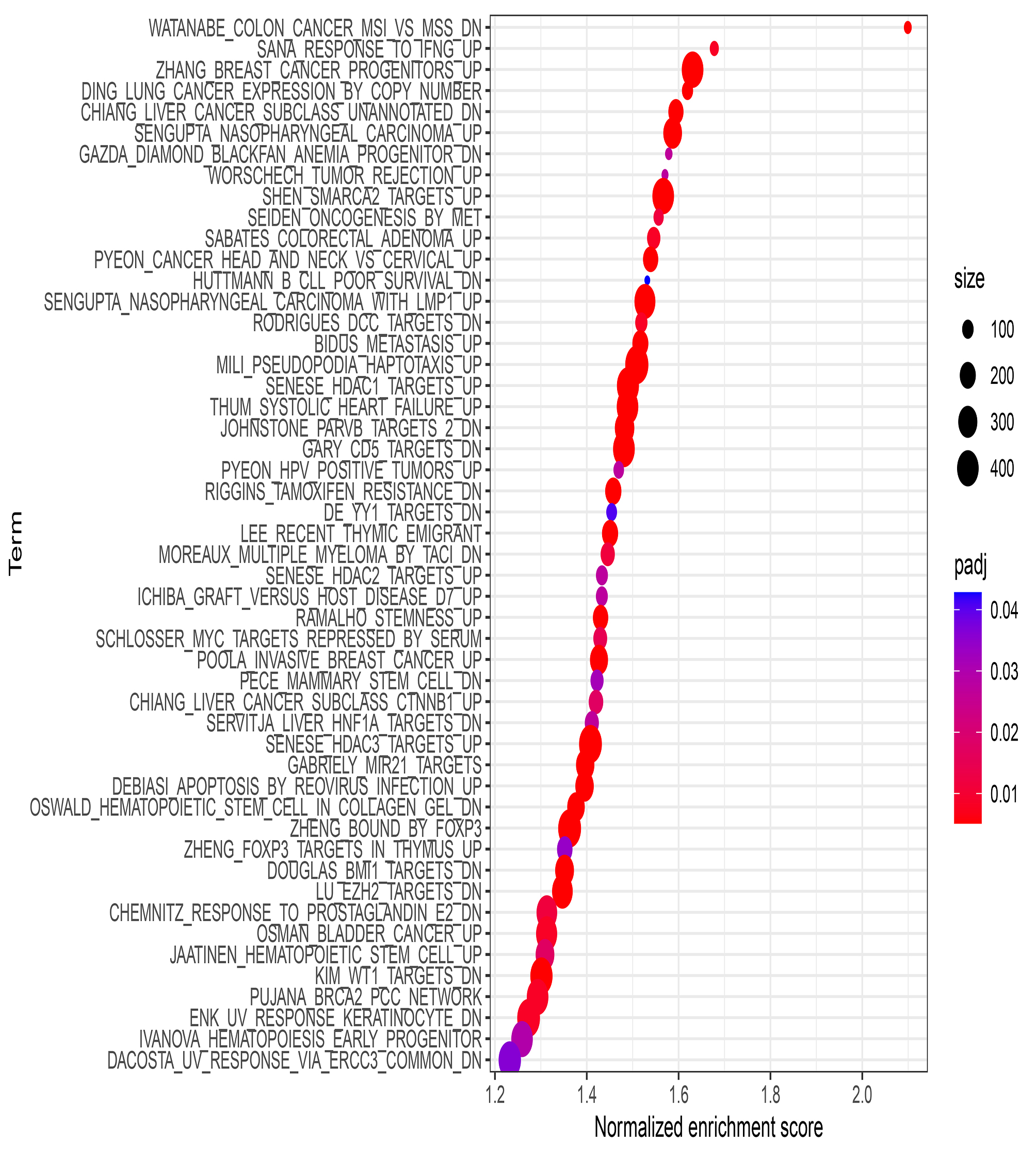

Supplement: Supplementary file 8 — Additional file 8: Figure S8. The expression characteristics of genetic perturbations significantly changed by the IRGPs model in GSE17538. [file 12885_2020_7532_MOESM8_ESM.jpg]

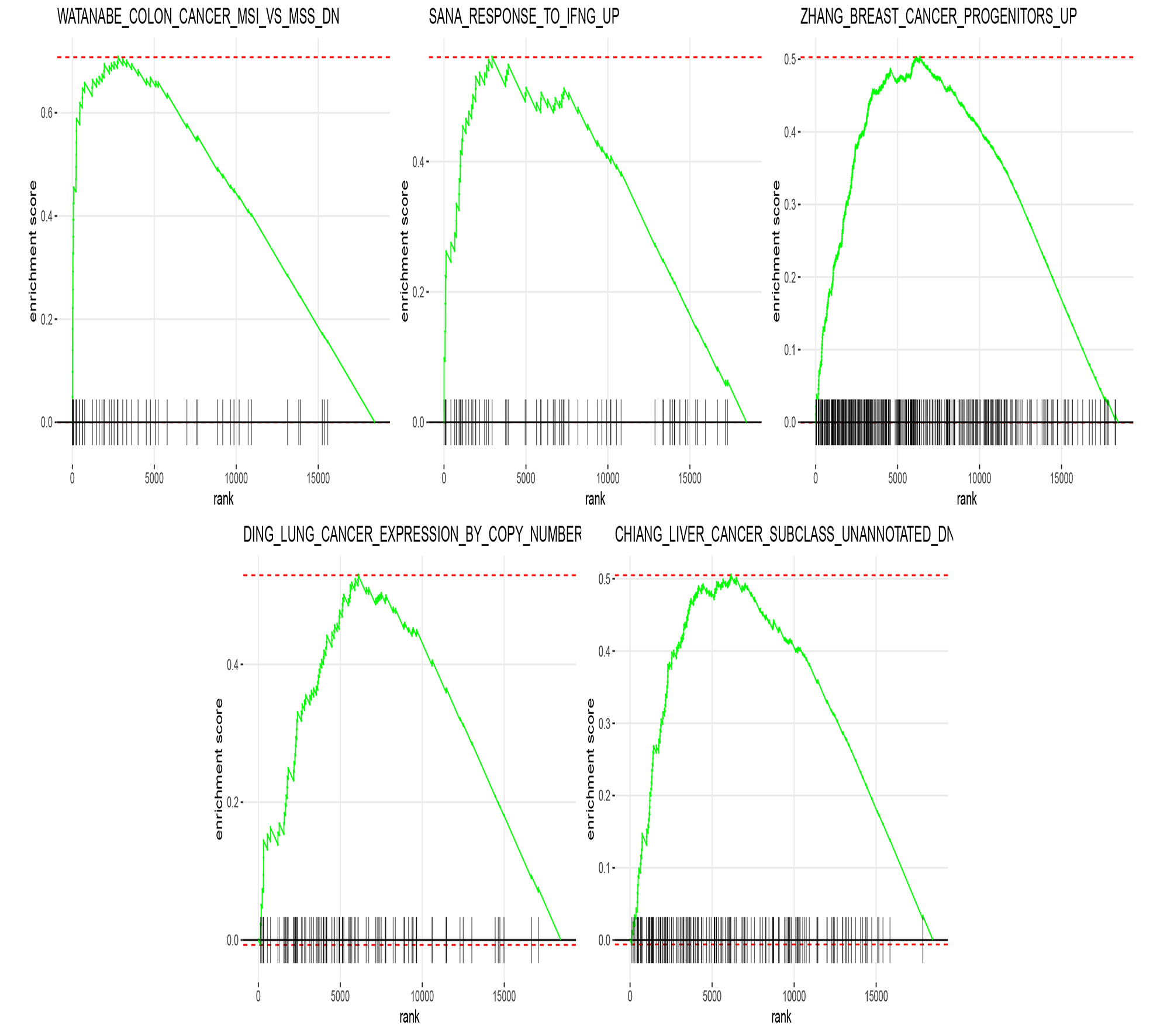

Supplement: Supplementary file 9 — Additional file 9: Figure S9. The top 5 results of GSEA in GSE17538. [file 12885_2020_7532_MOESM9_ESM.jpg]
